# Analysis of bone osteometry, mineralization, mechanical and histomorphometrical properties of tibiotarsus in broiler chickens demonstrates a influence of dietary chickpea seeds (*Cicer arietinum* L.) inclusion as a primary protein source

**DOI:** 10.1371/journal.pone.0208921

**Published:** 2018-12-11

**Authors:** Siemowit Muszyński, Ewa Tomaszewska, Piotr Dobrowolski, Małgorzata Kwiecień, Dariusz Wiącek, Izabela Świetlicka, Małgorzata Skibińska, Monika Szymańska-Chargot, Jolanta Orzeł, Michał Świetlicki, Marta Arczewska, Mariusz Szymanek, Mykola Zhyla, Monika Hułas-Stasiak, Halyna Rudyk, Agnieszka Tomczyk-Warunek

**Affiliations:** 1 Department of Physics, Faculty of Production Engineering, University of Life Sciences, Lublin, Poland; 2 Department of Animal Physiology, Faculty of Veterinary Medicine, University of Life Sciences in Lublin, Lublin, Poland; 3 Department of Comparative Anatomy and Anthropology, Faculty of Biology and Biotechnology, Maria Curie-Skłodowska University, Lublin, Poland; 4 Institute of Animal Nutrition and Bromathology, Faculty of Biology, Animal Science and Bioeconomy, University of Life Sciences in Lublin, Lublin, Poland; 5 Bohdan Dobrzański Institute of Agrophysics of the Polish Academy of Sciences, Lublin, Poland; 6 Department of Crystallography, Faculty of Chemistry, Maria Curie-Skłodowska University, Lublin, Poland; 7 Department of Radiochemistry and Colloid Chemistry, Faculty of Chemistry, Maria Curie-Skłodowska University, Lublin, Poland; 8 Department of Applied Physics, Faculty of Mechanical Engineering, Lublin University of Technology, Lublin, Poland; 9 Department of Agricultural, Horticultural and Forest Machinery, Faculty of Production Engineering, University of Life Sciences in Lublin, Lublin, Poland; 10 Laboratory of Clinical Biological Research, State Scientific Research Control Institute of Veterinary Medicinal Products and Feed Additives, Lviv, Ukraine; University of Illinois, UNITED STATES

## Abstract

This study was focused on analyzing the effects of dietary inclusion of raw chickpea seed as a replacement of soybean meal as a primary protein source on bone structure in broiler chickens. Broiler chickens (n = 160) received in their diet either soybean meal (SBM) or raw chickpea seeds (CPS) as a primary protein source throughout the whole rearing period (n = 80 in each group). On the 42^th^ day randomly selected chickens from each group (n = 8) were slaughtered. Collected tibiotarsus were subjected to examination of the biomechanical characteristics of bone mid-diaphysis, microstructure of the growth plate and articular cartilages; the analysis of mineral content and crystallinity of mineral phase, and the measurements of thermal stability of collagen in hyaline cartilage were also carried out. The inclusion of chickpea seeds resulted in increase of bone osteometric parameters (weight, length and mid-diaphysis cross-sectional area) and mechanical endurance (yield load, ultimate load, stiffness, Young modulus). However, when loads were adjusted to bone shape (yield and ultimate stress) both groups did not differ. Mineral density determined by means of densitometric measurements did not differ between groups, however the detailed analysis revealed the differences in the macro- and microelements composition. The results of FT-IR and XRD analyses showed no effect of diet type on mineral phase crystallinity and hydroxyapatite nanocrystallites size. In trabecular bone, the increase of real bone volume (BV/TV) and number of trabeculae was observed in the CPS group. Total thickness of articular cartilage was the same in both groups, save the transitional zone, which was thicker in the SBM group. The total thickness of the growth plate cartilage was significantly increased in the CPS group. The area of the most intense presence of proteoglycans was wider in the SBM group. The structural analysis of fibrous components of bone revealed the increase of fraction of thin, immature collagen content in articular cartilage, trabeculae and compact bone in the CPS group. The dietary inclusion of CPS affected the thermal stability of collagen, as decrease of net denaturation enthalpy was observed. This study showed a beneficial effect of CPS on the skeletal development, improving the overall bone development and the microarchitecture of cancellous bone. It suggests that CPS can be a promising replacement for SBM in broilers feeding in the aspect of animal welfare related to the development of the skeletal system.

## Introduction

Nutrition has been identified as a critical factor impacting skeletal growth and bone strength in poultry [1]. Among others, protein supply (quantity and source) is widely connected to bone development, remodeling, and mechanical strength [2, 3, 4].

Bone tissue consists of inorganic (mineral) constituents determining bone density and mechanical strength, while organic components form ossein, ensuring bone elasticity [1]. The greatest part of bone mineral structure is calcium and phosphorus located in hydroxyapatite crystallite structures. Ossein contains collagen fibres, proteoglycans, and other non-collagenous proteins. Bone remodeling and maturation, which involves changes in bone size and shape, depend on the interaction between bone cells activities, intermolecular networks of collagen, and interactions of proteoglycans and non-collagenous proteins. Poorly designed diet may influence these processes and lead to severe skeletal disorders [3, 5–9].

Soybean meal (SBM), a commonly used primary source of protein in feed, is often achieved from genetically modified (GM) varieties. Due to upcoming restrictions of using GM organisms in feeds, alternative sources of dietary protein are sought after. Moreover, as it is still not allowed in the EU countries to use meat and bone meal in livestock diets (EC directive 999/2001), the interest is focused on vegetable-based protein sources. The various vegetable-based protein sources have been tested, including distillers dried grains with solubles [10, 11], corn protein concentrate [12] or various grain legumes [13–15]. Among others, chickpea seeds (CPS) have been proposed as a possible SBM replacement [16, 17].

In our earlier work we have shown that the replacement of SBM with raw CPS in broiler chickens influenced mechanical properties and tendon collagen thermal properties of tendon [18]. As far as we know, there is no information in literature concerning the effects of CPS inclusion as a primary protein source on bone quality in poultry.

This study was designed to evaluate the effects of feeding broiler chickens with CPS as a replacement of SBM on tibiotarsus properties. The analyzed traits included assessment of bone densitometry and osteometric parameters, analysis of microelements content, comprehensive examination of the biomechanical characteristics of bone mid-diaphysis and microstructure of the growth plate and articular cartilages, analysis of crystallinity of mineral phase, and measurements of thermal stability of collagen in hyaline cartilage. Applied techniques allow to provide detailed information about the material composition and structural organization of both inorganic and organic bone components and can help to better understand how they contribute to the bone mechanical properties.

## Material and methods

All procedures conducted with the chickens had been prior approved by the 2nd Local Ethics Committee for Animal Testing at the University of Life Sciences in Lublin, Poland (33/2015). This study was carried out in strict accordance with the recommendations of the National Ethic Commission (Warsaw, Poland).

### Experimental design

A total of 160, one-day old, male broiler chickens (Ross 308), obtained from a local commercial hatchery, were used in this experiment. The chicks (initial weight 38.8±1.1 g) were randomly allocated into two group (n = 80 in each), fed either soybean meal (the SBM group) or raw chickpea seeds (the CPS group) as a primary protein source throughout the whole rearing period (Table 1). The diets, iso-nitrogenous, iso-protein, and isoenergetic, were formulated to meet or exceed the nutritional requirement [19]. Bird management and care are described in details in [18]. Individual body weight and feed intake (per pen) were monitored. At 42^nd^ day 8 birds from each group were stunned using the method of electrical stunning and then decapitated. Immediately after slaughter, tibiotarsus from individual chickens were dissected, scraped away from any adhering tissues, weighted, wrapped in gauze soaked in saline and kept frozen at -25°C until further examination. Samples of articular cartilage for thermal analyses were collected before freezing. In subsequent stages of analyses, the right tibiotarsus was subjected to strength tests, while the bone collected from the left side of the chicken was used for osteometric measurements, densitometry, and histomorphometric analysis.

**Table 1:** Composition and nutritive value of the diets fed during the trial.

Ingredient (%)	Starter (days 1–21)		Grower (days 22–35)		Finisher (days 36–42)	
	SBM	CPS	SBM	CPS	SBM	CPS
Maize	10.00	10.00	10.00	10.00	15.00	10.00
Wheat	53.75	21.40	44.91	19.41	35.25	19.95
Soybean meal^1^	28.65		21.50	-	19.40	-
Chickpea seeds^2^	-	45.00	-	45.00		45.00
Triticale	-	10.00	10.00	10.00	15.00	10.00
Rapeseed meal	-	2.00	4.00	-	5.00	
Soybean oil	2.40	2.40	4.40	4.40	5.20	5.20
Monocalcium phosphate	0.88	0.88	0.83	0.83	0.80	0.80
Limestone	1.35	1.35	1.31	1.31	1.30	1.30
Sodium bicarbonate	0.08	0.08	0.08	0.08	0.08	0.08
Sodium chloride	0.30	0.30	0.27	0.27	0.27	0.27
Fat-protein concentrate^3^	1.00	1.00	1.00	1.00	1.00	1.00
Premix vita-min	0.50^I^	0.50^I^	0.50^II^	0.50^II^	0.50^III^	0.50^III^
Choline chloride	-	4.00	-	6.00	-	4.70
DL-methionine 99%	0.09	0.09	0.10	0.10	0.10	0.10
L-lysine HCl 78%	0.30	0.30	0.30	0.30	0.30	0.30
L-threonine 99%	0.50	0.50	0.50	0.50	0.50	0.50
Carbovet^4^	0.20	0.20	0.30	0.30	0.30	0.30
Nutritional value of 1 kg mixture:						
^a^ Metabolizable energy, MJ/kg	12.4	12.5	12.9	13.0	13.1	13.1
^b^ Crude protein, %	21.1	21.2	19.0	19.1	18.0	18.1
^b^ Crude fat, %	4.28	5.21	6.23	8.23	7.09	9.0
^b^ Crude fiber, %	3.12	1.32	3.34	1.23	3.37	1.24
^b^ Lysine, %	1.34	0.98	1.21	0.86	1.14	0.78
^b^ Methionine + Cysteine, %	0.97	0.82	0.88	0.65	0.90	0.61
^b^ Total calcium, %	0.93	0.83	0.91	0.82	0.82	0.81
^b^ Total phosphorus, %	0.69	0.51	0.69	0.45	0.68	0.44
^a^ Bioavailable phosphorus, %	0.44	0.35	0.42	0.34	0.41	0.33
^a^ Total Ca / bioavailable P	2.12	2.32	2.14	2.40	2.17	2.41

^1^ 46% crude protein in dry matter

^2^ 21% crude protein in dry matter

^3^ 1 kg of fat-protein concentrate contains: 39% crude protein, 2% crude fat, 10.8 MJ metabolizable energy

^4^ 90% airy charcoal in dry matter

^I^ The premix provided per 1 kg of starter: Mn 100 mg, Fe 40 mg, Cu 16 mg, I 1 mg, Se 0.15 mg, vitamin A 15 000 IU, vitamin B_1_ 3 mg, vitamin B_2_ 8 mg, vitamin B_6_ 5 mg, vitamin B_12_ 0.016 mg, vitamin D_3_ 5 000 IU, vitamin E 75 mg, vitamin K_3_ 4 mg, choline 1 800 mg, folic acid 2 mg, biotin 0.2 mg, nicotinic acid 60 mg, pantothenic acid 18 mg

^II^ The premix provided per 1 kg of grower: Mn 100 mg, Fe 40 mg, Cu 16 mg, I 1 mg, Se 0.15 mg, vitamin A 12 000 IU, vitamin B_1_ 2 mg, vitamin B_2_ 6 mg, vitamin B_6_ 4 mg, vitamin B_12_ 0.016 μg, vitamin D_3_ 5 000 IU, vitamin E 50 mg, vitamin K_3_ 3 mg choline 1 600 mg, folic acid 1.75 mg, biotin 0.2 mg, nicotinic acid 60 mg, pantothenic acid 18 mg

^III^ The premix provided 1 per kg of finisher: Mn 100 mg, Fe 40 mg, Cu 16 mg, I 1 mg, Se 0.15 mg, vitamin A 12 000 IU, vitamin B_1_ 2 mg, vitamin B_2_ 5 mg, vitamin B_6_ 3 mg, vitamin B_12_ 0.011 μg, vitamin D_3_ 5 000 IU, vitamin E 50 mg, vitamin K_3_ 2 mg, choline 1 600 mg, folic acid 1.5 mg, biotin 0.05 mg, nicotinic acid 35 mg, pantothenic acid 18 mg

^a^ calculated values

^b^ analyzed values

### Bone mechanical testing

The mechanical properties of bones were determined using the three-point bending test performed on a universal testing machine (Zwick Z010, Zwick-Roell GmbH & Co., Ulm, Germany) after overnight thawing of bone. The bone was loaded in the anterior-posterior plane with a displacement rate of 10 mm/min until fracture. The support span was 40% of the bone length [20, 21]. The yield load, ultimate load and stiffness, indicating bone structural properties, were determined from the force-displacement curves recorded during the test [22] using the Origin 2016 software (OriginLab, Northampton, MA, USA). Whole-bone material properties (Young modulus, yield stress, yield strain, ultimate strain, and ultimate stress) were calculated using standard engineering beam-theory equations as previously described [23].

### Osteometric measurements

Bone mid-diaphysis cross-sectional geometry was determined on the basis of measurements of external and internal diameters of the mid-diaphysis cross-sectional (both in medial-lateral and anterior-posterior plane). The calculated geometric properties were: cortical cross section area, cortical index, mean relative wall thickness, radius of gyration and cross-sectional moment of inertia [23, 24]. The Seedor index (the ratio of bone weight and length) was also calculated.

### Histomorphometric analysis

The sagittal sections of proximal end of tibiotarsus containing articular and growth plate cartilages, trabecular and compact bone were cut off from the middle of the lateral condyle and subjected to histology and microscopy procedure according to previously described methodology and equipment [8, 9]. Safranin O staining was employed for the visualization of proteoglycans and to the visual assessment of Mankin scoring system which was used to evaluate articular cartilage. Goldner’s trichrome staining was used to assess the morphology of the growth plate cartilage and the articular cartilage. The thickness of the main zones of the growth plate cartilage: reserve zone (the zone I), proliferation zone (the zone II), hypertrophy zone (the zone III) and calcification zone (the zone IV) was measured at four sites along the cartilage as described previously [25]. Similarly, the thickness the following zones of the articular cartilage was measured: horizontal zone (superficial surface, the zone I), transitional zone (the zone II) and radial zone (the zone III) [26]. The Picrosirius red (PSR) staining was used to evaluate the distribution of thin, immature, and thick, more mature collagen fibres in articular cartilage, trabecular and compact bone [27, 28]. The trabecular bone morphometry was measured on the microscopic images using ImageJ software (Wayne Rasband, NIMH, Bethesda, MD, USA). The calculated morphometric parameters were: relative bone volume (BV/TV), mean trabecular separation (Tb.Sp mean), mean trabecular thickness (Tb.Th mean) and number of trabeculae (Tb.N) [29].

### Measurements of articular cartilage thermal stability

Thermal analysis of articular cartilage was performed to examine the structural changes in collagen. Samples of articular cartilage obtained from the middle of lateral condyle were washed in distilled water, dried superficially, placed in the 40 μl aluminum DSC pans, and sealed to prevent moisture loss. Thermal analysis was performed with a DSC-1 calorimeter (Mettler-Toledo GmbH, Switzerland) from 20 °C to 90 °C with a heating rate of 10 °C/min and an empty pan as a reference [30]. The onset temperature (T_onset_), temperature of maximum heat absorption (T_peak_) and net enthalpy of the denaturation process (ΔH) were determined from the thermograms using a software integrated with the calorimeter. After the calorimetry, punctured pans were dried for 24 h at the temperature of 105 °C. The denaturation enthalpies ΔH were normalized to samples dry weights [18].

### Bone mid-diaphysis mineral density, volumetric density and ash content

Analyzes were performed for the bone mid-diaphysis covering the fragment of bone subjected to strength tests. Before the measurements, the bone marrow was removed from bone mid-diaphysis and the bones were defatted using the following procedure: (1) removing bone marrow under running water; (2) defatting in 50/50 acetone/ethanol mixture with agitation for 24 h; (3) removing marrow residues with a high-pressure water jet (using a syringe). The analysis of mineral density was performed using the dual-energy X-ray absorptiometry (DXA) method on a Discovery W densitometer (Hologic Inc., Marlborough, MA, USA). The measurement of bone volumetric density was performed with a helium gas pycnometer as described previously [31, 32]. Next, the samples were calcined in a muffle furnace at 500 °C for 24 h to determine the ash percentage which was expressed relative to the sample dry weight.

### Bone macro- and microelements content

The composition of bone mineral phase was determined using ICP-OES spectrometry (iCAP Series 6500, Thermo Scientific, Waltham, MA, USA) in ashed bone mid-diaphysis samples. The TraceCERT multi-element stock solution (Sigma-Aldrich, St. Louis, MO, USA) was used to prepare reference standards. The macro- and microelements content in samples were expressed mg or μg in 1 g of crude ash.

### Structural analysis of bone mineral phase

FT-IR spectra of ashed bone samples were collected using a Nicolet 6700 spectrometer (Thermo Scientific, Waltham, MA, USA) over the range 4000–650 cm^-1^. Baseline corrections were performed using Omnic software (Thermo Scientific, Waltham, MA, USA).

The crystallinity of bone mineral phase was measured XRD method using Empyrean X-ray diffractometer (PANalytical, Almelo, The Netherlands). Samples were measured in θ-2θ geometry over a range from 10 to 80 deg with step size of 0.01 deg and counting time 6 s per data point [22]. The mean size of the nanocrystallites was calculated according to the Scherrer equation [33] with the shape constant of 0.9 and apparatus broadening of 0.01 deg. Miller indices (200), (3–10), and (300) were taken for the calculation of nanocrystallites size in *a-b* plane, the size in *c* direction was calculated on the basis of indices (002) and (004) [34]. Bragg peaks and crystallographic directions were identified using Mercury CSD 3.10.1 software (CCDC, Cambridge, UK) from the hydroxyapatite references (No. 2300273, Crystallography Open Database; No. 96-901-0053, High Score Plus package software). The peak position and FWHM (*full width at half maximum*) were calculated from the fits of the Voight function to every peak using Origin 9.0 software.

### Statistical analysis

An individual bird was considered as the experimental unit. The normality of data distribution was tested using the Shapiro—Wilk test. A comparison between normally distributed data was carried out using Student’s t test. When data were not normally distributed the Mann—Whitney U test was applied. For all tests a P < 0.05 was considered statistically significant. The data were analyzed using Statistica 13 software (TIBCO Software Inc., Palo Alto, CA, USA).

## Results

The type of diet did not affect growth rate or feed efficiency of broilers. The broilers weighed on average 2320±125 g in the SBM group and on average 2404±170 g in the CPS group (P > 0.05) [18]. Also feed conversion ratio (FCR) was not significantly influenced by dietary treatment (1.93±0.34 kg/kg and 1.88±0.44 kg/kg, in the SBM and the CPS groups, respectively, P > 0.05).

**Fig 1:**
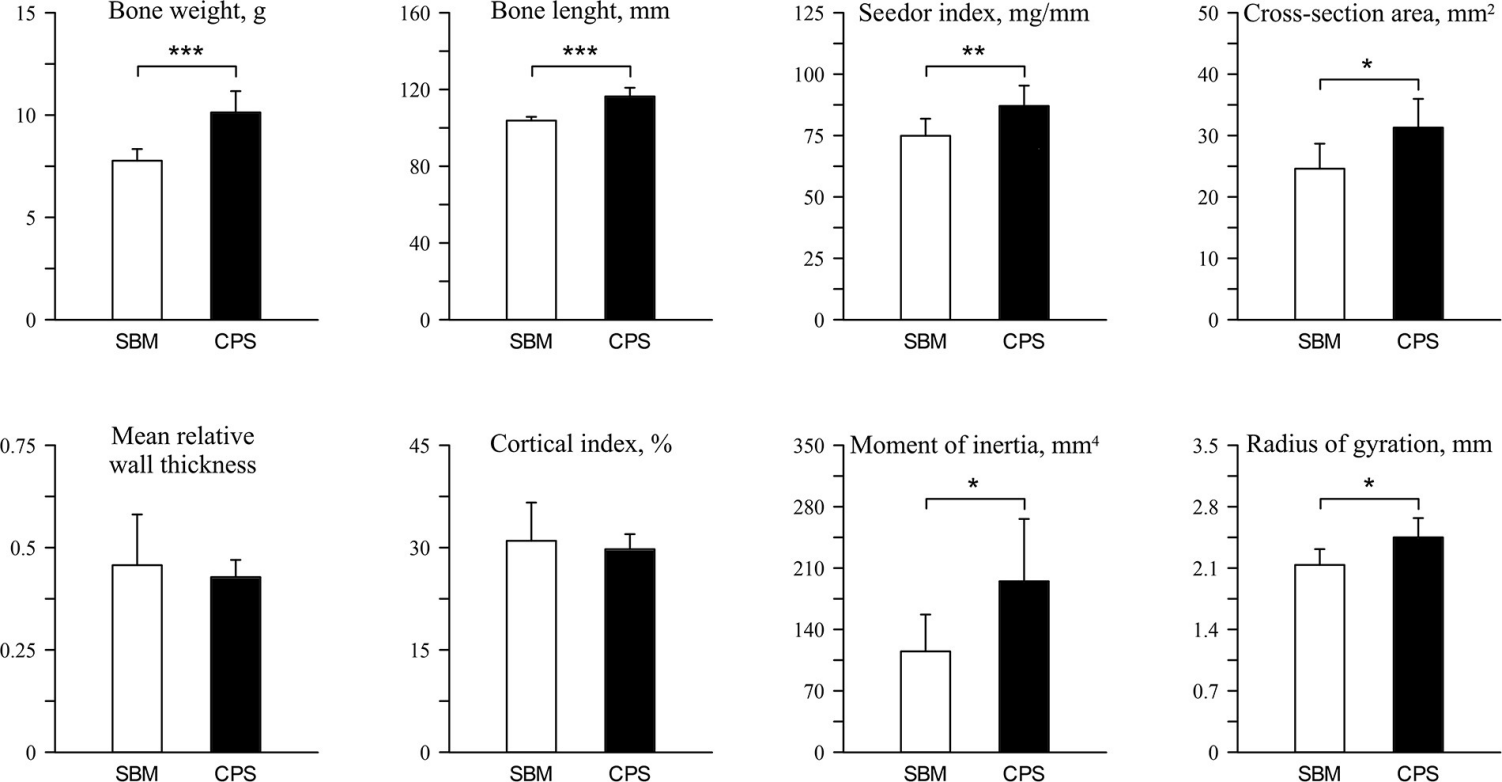
Geometric characteristics of tibiotarsus of 42 days-old broiler chickens fed soybean meal (SBM) or chickpea seeds (CPS) as a primary protein source.

The diet type have a significant effect on the weight and length of the bones (Fig 1) which were significantly heavier and longer in the CPS group (increase of 30% and 12%, respectively; P < 0.001 for both values). Also the Seedor index was significantly higher in the CPS group (P < 0.01). From the indices describing bone mid-diaphysis geometry only cross-sectional area significantly increased (P < 0.01). Nevertheless, the change in the spatial distribution of bone tissue influenced the values of the cross-sectional moment of inertia and radius of gyration which were significantly increased in the CPS group (increase for 70% and 15%, respectively; P < 0.05 for both values).

**Fig 2:**
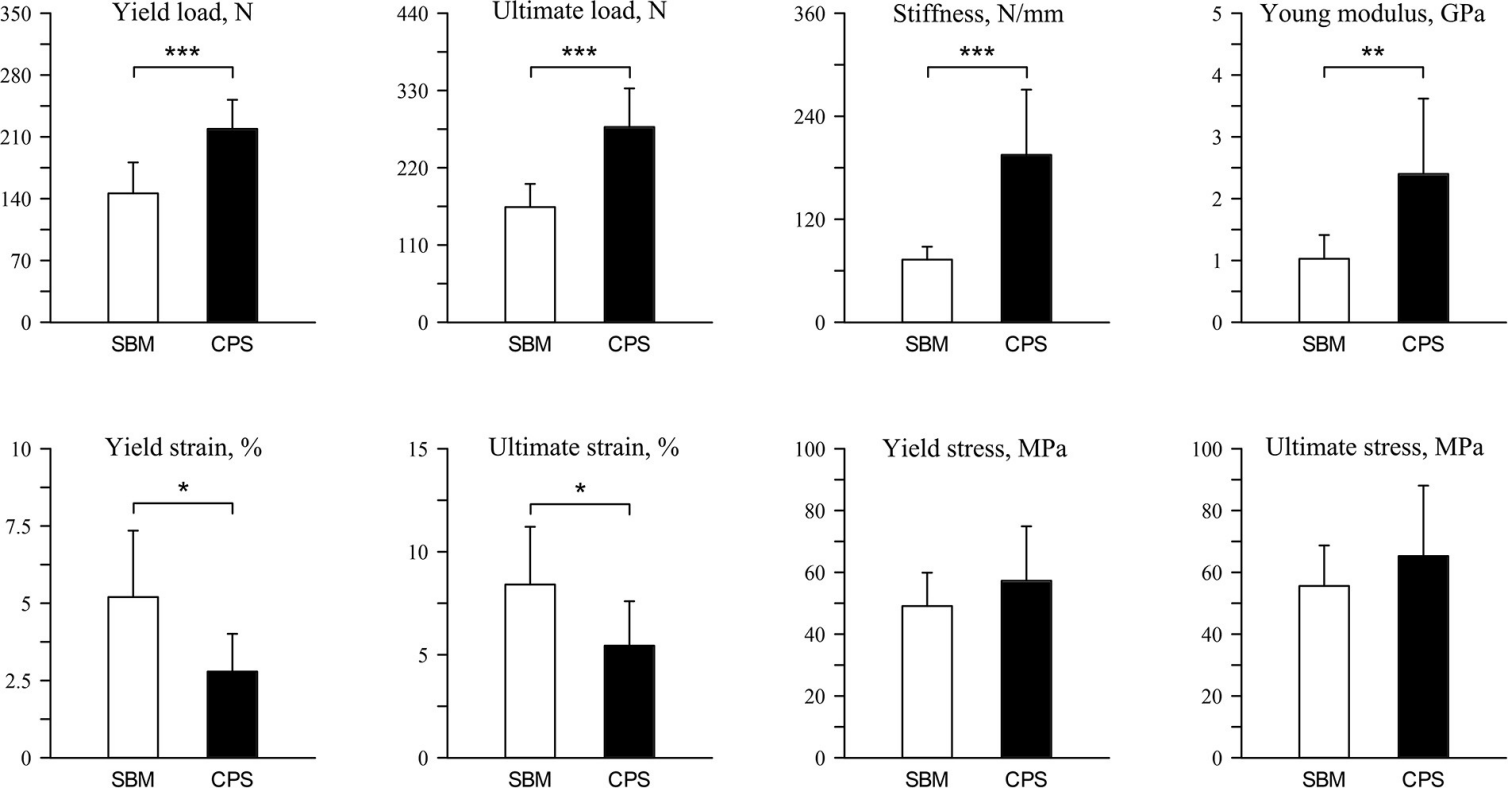
Mechanical properties of tibiotarsus mid-diaphysis of 42 days-old broiler chickens fed soybean meal (SBM) or chickpea seeds (CPS) as a primary protein source.

The dietary inclusion of CPS influenced bone mechanical strength. Yield load increased by 50% and ultimate load by 70% when compared to the SMB group (Fig 2, P < 0.001 for both values). Similarly, a statistically significant increase of bone stiffness and Young modulus were observed (P < 0.001 and P < 0.01, respectively). On contrary, yield strain and ultimate strain were significantly lower in the CPS group (decrease by 50%, and 35%, respectively; P < 0.05 for both values). The other mechanical parameters (ultimate strain, yield stress and ultimate stress) were not influenced by the diet type.

The ash percentage was the same in both groups (Table 2) while bone volumetric density was significantly lower in the CPS group (by 8%, P < 0.001). The mineral density determined by means of DXA measurements did not differ between groups, however the ICP-OES analysis revealed the differences in the macro- and microelements composition (Table 2). The content of Ca was higher in the CPS group (P < 0.05). Therefore, as P content was not affected by the diet, an increase (P < 0.05) of the Ca/P ratio in the CPS group was observed. Also the Mn and Sr content and Sr/Ca ratio were higher (P < 0.05, P < 0.01, and P < 0.05, respectively) while the content of S was lower in the CPS group (P < 0.001).

**Table 2:** Densitometry properties, macro- and microelements content in tibiotarsus mid-diaphysis.

Dependent variable	group		P value
	SBM	CPS	
*Density and ash content*			
Volumetric density, g/cm^3^	1.73±0.03	1.58±0.06	***
Mineral density, g/cm^2^	0.140±0.030	0.156±0.058	n.s.
Ash, %	55.7±1.6	53.8±1.2	n.s.
*Macro- and microelements content*			
Ca, mg/g	329±5	337±6	*
Cd, μg/g	0.074±0.030	0.074±0.025	n.s.
Co, μg/g	0.219±0.037	0.291±0.106	n.s.
Cr, μg/g	7.06±1.25	6.28±0.38	n.s.
Cu, μg/g	7.39±1.20	6.47±3.32	n.s.
Fe, μg/g	190±29	152±39	*
Li, μg/g	38.9±3.5	39.7±4.3	n.s.
Mg, mg/g	6.84±0.34	6.71±0.31	n.s.
Mn, μg/g	5.69±0.82	7.49±1.59	*
Ni, μg/g	3.10±1.77	2.20±0.75	n.s.
P, mg/g	148±1	149±2	n.s.
Pb, μg/g	2.19±0.74	2.31±0.76	n.s.
S, mg/g	3.18±0.16	2.72±0.21	***
Se, μg/g	0.341±0.190	0.362±0.164	n.s.
Si, μg/g	87.0±15.0	92.2±9.5	n.s.
Sr, μg/g	160±42	225±12	**
Zn, μg/g	289±11	304±20	n.s.
Sr/Ca, g/kg	0.471±0.111	0.613±0.131	*
Ca/P, g/g	2.22±0.02	2.27±0.04	*

Values are presented as mean ± standard deviation.

* P < 0.05;

** P < 0.01,

*** P < 0.001; n.s.–not significant.

**Fig 3:**
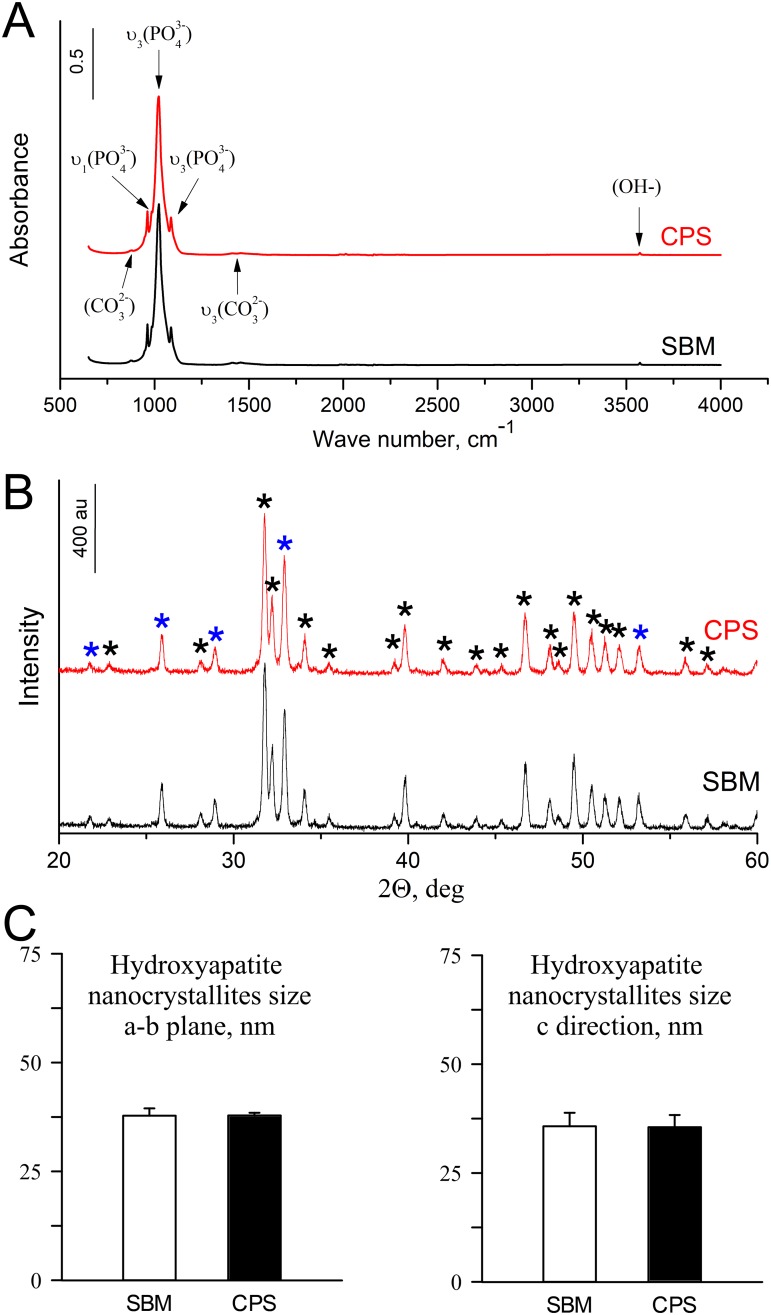
The structural (FT-IR and XRD spectra) analysis of SBM and CPS tibiotarsus mid-diaphysis samples calcined at 500 °C. (A) In FT-IR spectra both samples show strong two visible bands (at ∼1023 and ∼1089 cm^-1^) indicating the *v3* stretching mode of phosphate anion (PO_4_^3−^) vibrations, characteristic of the hydroxyapatite [34]. Also peak produced by the ν*1* symmetric stretching vibrations of the P-O mode of phosphate (PO_4_^3−^) at ∼962 cm^−1^ indicate the presence of pure mineral phase, free from organic matter [34]. The weak peaks at ∼876 and ∼1438 cm^−1^ correspond to the CO_3_^2−^ functional group. Carbonate ions are a common impurity in bone hydroxyapatite [35]. Finally, the weak peak located at 3570 cm^−1^ corresponds to the vibrations of OH− ions in the hydroxyapatite lattice [36]. (B) The crystalline nature and purity of calcified bone samples have been confirmed by XRD analysis. The XRD peaks, marked with asterisk (*) at the CPS diffractogram, were found to be in good conformity with that of the hydroxyapatite standard (96-901-0053, High Score Plus package software) in both groups. The peak position and the FWHM values of the most characteristic peaks are shown in Table 3. The peaks marked with blue asterisk were used for calculations of hydroxyapatite nanocrystallites size. (C) Calculated bone hydroxyapatite nanocrystallites size in *a-b* plane, and in *c* direction.

The results of FT-IR and XRD analyses presented in Fig 3 and Table 3. In both groups mineral phase showed FT-IR and XRD spectra typical for hydroxyapatite structures. There was no effect of diet on bone hydroxyapatite nanocrystallites size (Fig 3C, Table 3).

**Table 3:** The position and FWHM (full width at half maximum) of the most characteristic XRD peaks of hydroxyapatite structure in analyzed bone samples.

Reference standard^1^ peak position (deg)	Peak position (deg)		Peak FWHM	
	SBM	CPS	SBM	CPS
25.87	25.86	25.87	0.195	0.197
31.76	31.76	31.75	0.195	0.201
32.18	32.18	32.18	0.181	0.181
32.90	32.89	32.88	0.202	0.227
34.05	34.04	34.04	0.180	0.190
39.79	39.79	39.78	0.179	0.213
46.69	46.68	46.68	0.211	0.216
49.47	49.47	49.47	0.191	0.211

^1^ Reference standard: Apatite-(CaOH), #96-901-0053, High Score Plus package software.

**Fig 4:**
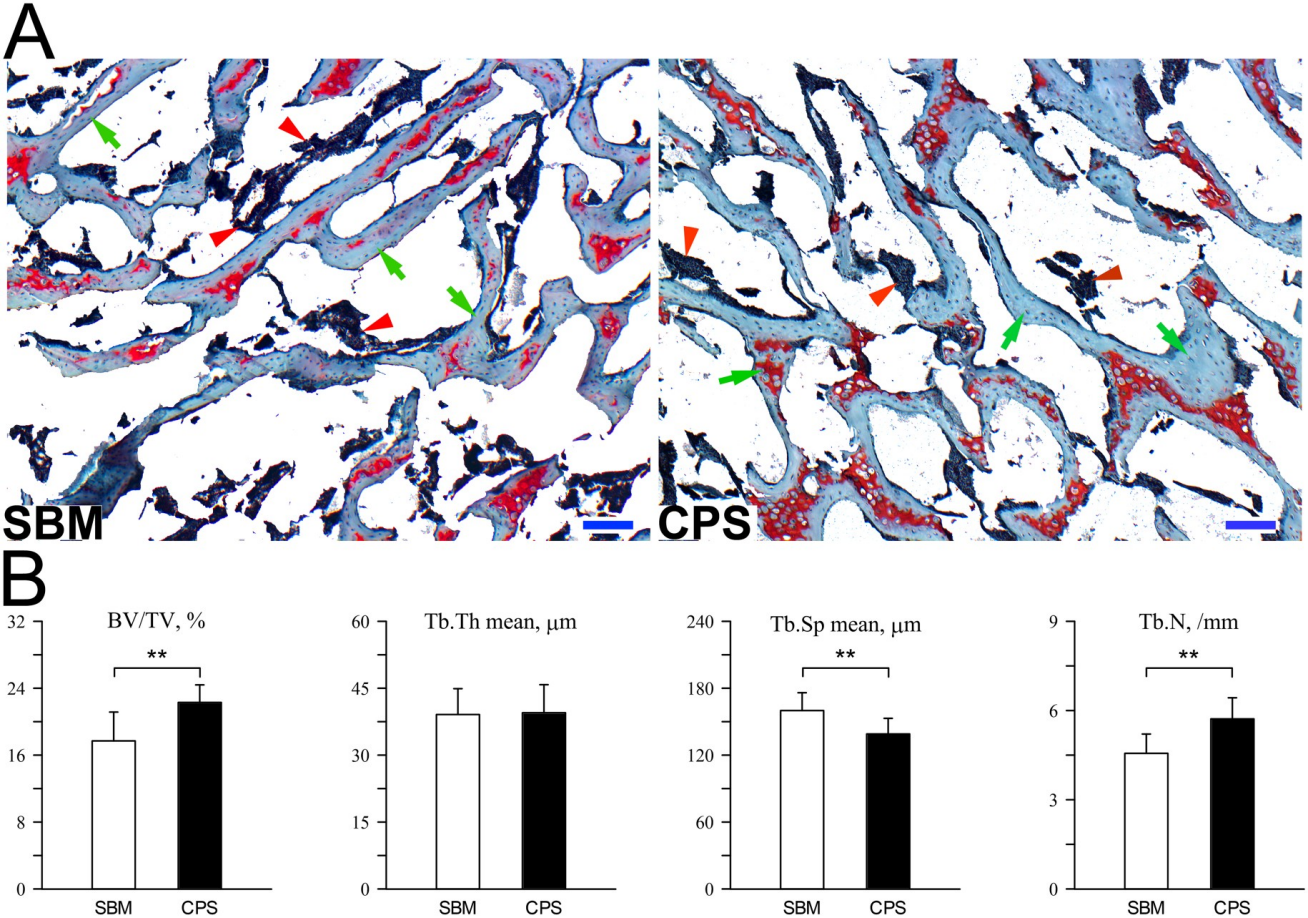
Histomorphometry of cancellous bone in tibiotarsus proximal part of 42 days-old broiler chickens fed soybean meal (SBM) or chickpea seeds (CPS) as a primary protein source. (A) Representative images of Goldner’s trichrome staining carried out on formaldehyde-fixed sections of trabecular bone. Green arrows indicate trabeculae, green arrowhead indicate bone marrow. All scale bars represent 50 μm. (B) The real bone volume (BV/TV) and number of trabeculae (Tb.N) significantly increased when CPS was introduced to the diet. A significant decrease of mean trabecular space (Tb.Sp mean) was additionally observed in the CPS group.

Representative microscopic images of trabeculea of cancellous bone in tibiotarsus and calculated histomorphometrical parameters are presented in Fig 4. The real bone volume (BV/TV) significantly increased in the CPS group (P < 0.01) which is a consequence of more numerous trabeculae (Tb.N, P < 0.01) and lower trabecular space (Tb.Sp mean, P < 0.01) as no changes in the mean trabecular thickness (Tb.Th mean) were observed.

The diet did not influence the total thickness of the of articular cartilage and thickness of its zones, except the zone II which was thicker in the SBM group (Table 4; P < 0.001). The total thickness of the growth plate cartilage was significantly increased in the CPS group (by 20%, P < 0.001; Table 4). This resulted from the thickening of the zone IV (by 45%, P < 0.001) after dietary CPS inclusion as the thicknesses of the zone I and zone III decreased (P < 0.001 and P < 0.01, respectively). The thickness of the zone II in growth plate cartilage was similar in both groups.

**Table 4:** Total thickness and thicknesses of particular zones in articular cartilage and growth plate cartilage.

Dependent variable	group		P value
	SBM	CPS	
*Articular cartilage*			
Zone I, μm	149±32	141±45	n.s.
Zone II, μm	465±127	272±123	***
Zone III, μm	777±210	702±208	n.s.
Total thickness, μm	2330±102	2440±177	n.s.
*Growth plate cartilage*			
Zone I, μm	271±86	145±29	***
Zone II, μm	270±125	258±68	n.s.
Zone III, μm	538±140	433±109	**
Zone IV, μm	896±361	1307±356	***
Total thickness, μm	1973±543	2373±572	***

Values are presented as mean ± standard deviation.

* P < 0.05;

** P < 0.01,

*** P < 0.001; n.s.–not significant.

**Fig 5:**
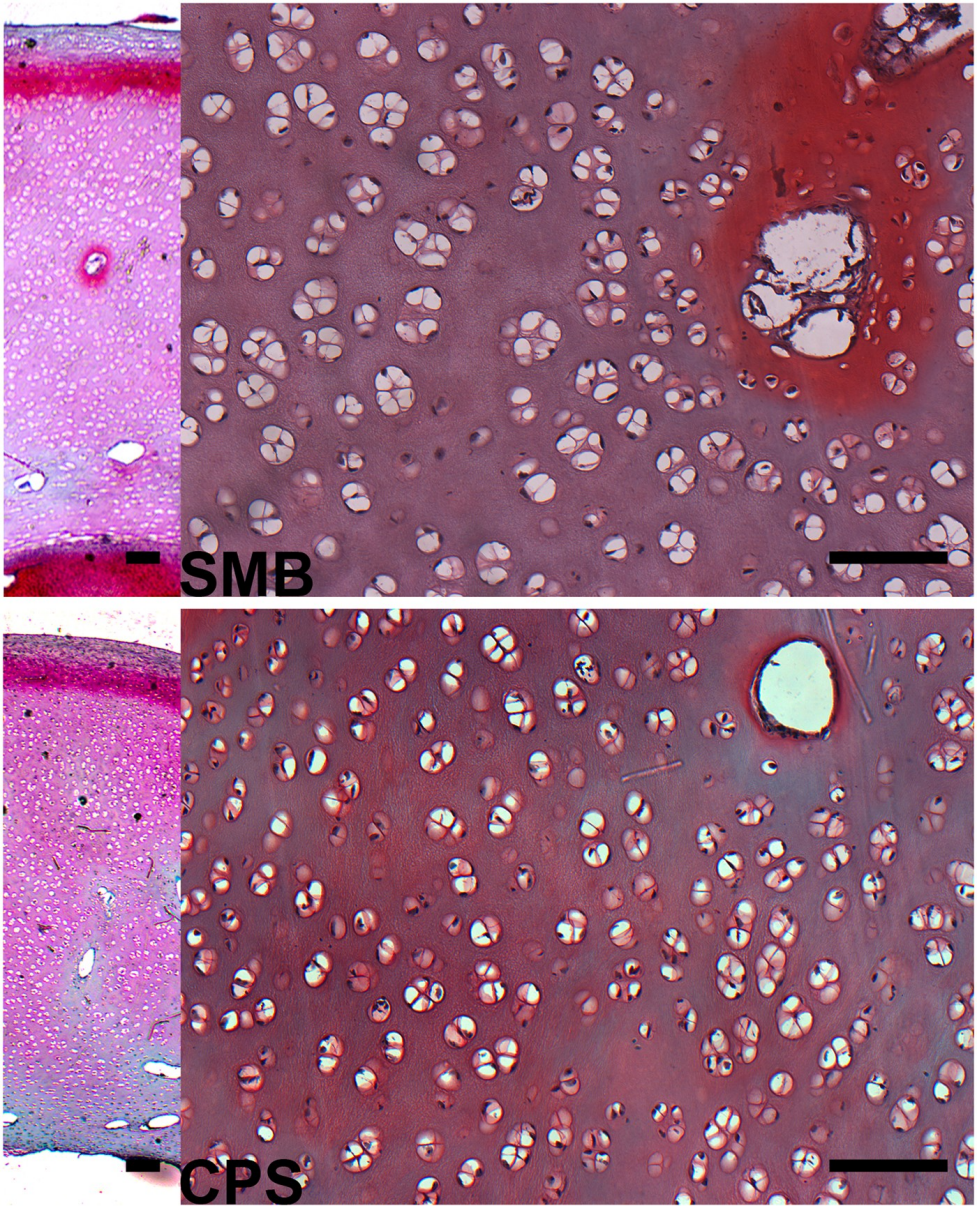
Representative light images of safranin O stained formaldehyde-fixed sections of tibiotarsus articular cartilage of 42 days-old broiler chickens fed soybean meal (SBM) or chickpea seeds (CPS) as a primary protein source. Left: Vertical sections of tibiotarsus articular cartilage. In both groups, the most intense staining pattern with safranin O indicating proteoglycans presence was observed in beginning of transitional zone, however, area of the highest proteoglycan concentration was wider in the SBM group. Right: Strong red color was tightly around isogenous groups of chondrocytes and nutritional ducts in pericellular matrix. All the scale bars represent 50 μm.

Proteoglycans content in the articular cartilage in the extracellular matrix was the same in both groups. The most intense staining pattern with safranin O indicating proteoglycans presence was observed in the beginning of the zone II. However, the area of this staining was broadened in the SBM group (Fig 5). In both groups the surface of the articular cartilage was smooth without irregularities according to Mankin score system (Fig 5). Also the osteochondral junction was intact in both groups.

**Fig 6:**
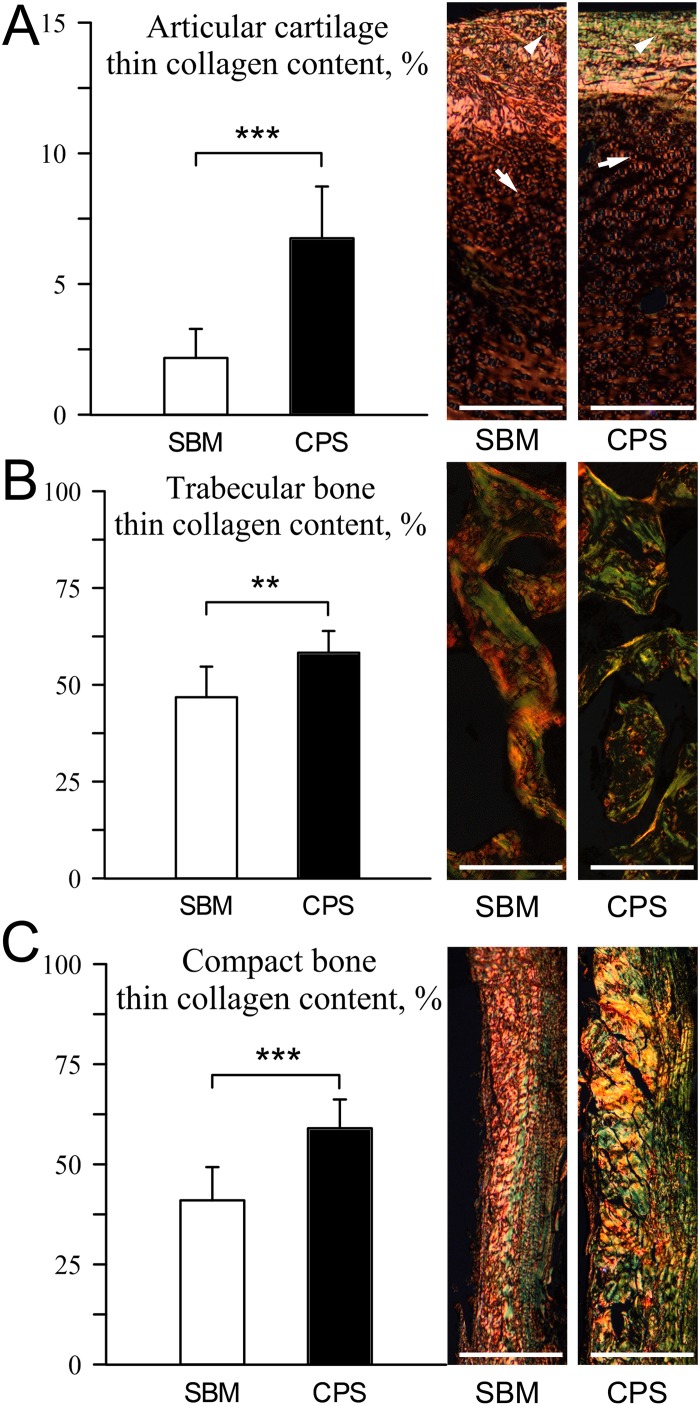
Quantitative analysis of presence of thin, immature collagen content in formaldehyde-fixed sections of articular cartilage, trabecular bone, and compact bone of tibiotarsus of 42 days-old broiler chickens fed soybean meal (SBM) or chickpea seeds (CPS) as a primary protein source. Representative polarized light images of PSR staining and determined percentages of thin, immature collagen fibres of bone tissues: (A) articular cartilage, (B) trabecular bone, (C) compact bone. Thicker, well-organized and more mature collagen fibres show orange-red birefringence (arrows) and the thinner ones, including reticular fibers, are green (arrowheads). All the scale bars represent 50 μm.

The structural analysis of fibrous components in PSR-stained sections of bone revealed the increase of fraction of thin, immature (green) collagen content in all examined tissues in the CPS group: articular cartilage, trabeculae, and compact bone (Fig 6A, 6B and 6C; P < 0.001, P < 0.01 and P < 0.001, respectively).

**Fig 7:**
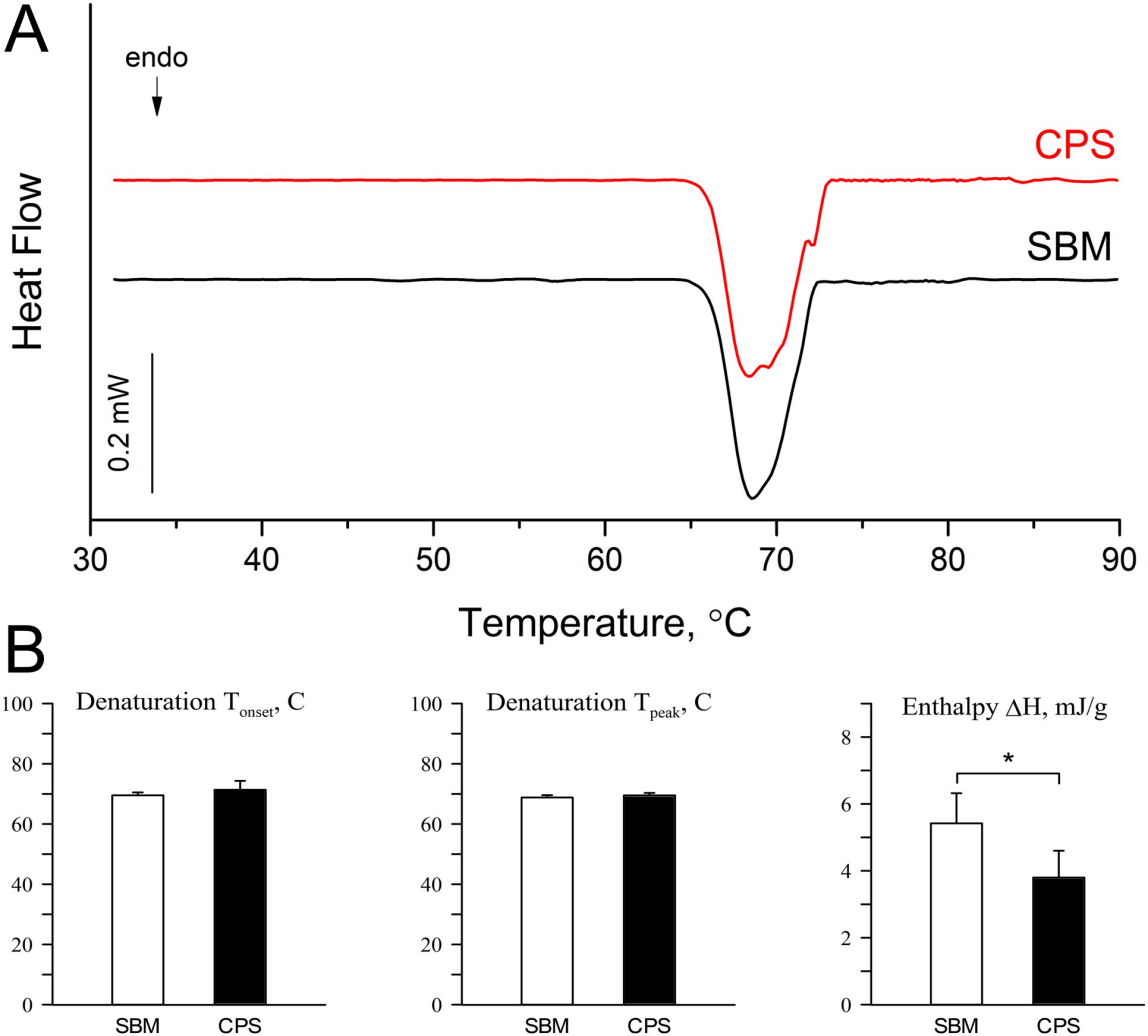
Thermal analysis of collagen structure in articular cartilage of 42 days-old broiler chickens fed soybean meal (SBM) or chickpea seeds (CPS) as a primary protein source. (A) The example DSC thermograms of thermal analysis performed on the cartilage samples from both groups. The endotherm peak indicates the denaturation of cartilage collagen. (B) The quantitative analysis of thermal denaturation of cartilage collagen.

The result of DSC analysis is shown in Fig 7. Both samples show single, endothermic peak linked with irreversible denaturalization of collagen proteins (Fig 7A). The dietary inclusion of CPS affected the thermal stability of collagen, as decrease of net denaturation enthalpy in the CPS group was observed (Fig 7B, P < 0.05). However, onset and peak denaturation temperatures did not differ between dietary treatments.

## Discussion

Bone mineral phase, organized in forms of hydroxyapatite crystals, contributes mainly to bone fracture strength and stiffness [35, 37]. Bone organic matrix is responsible not only for bone elastic properties, but also contributes to overall bone integrity, as it provides structural scaffolding to the inorganic phase [1, 38]. Thus, in order to properly assess the mechanical properties of bone, it is necessary to analyze the structure, organization and characteristics of both bone inorganic and organic components. There is little information regarding the changes in organic and inorganic components in the context of dietary protein [1] and results only of a few studies on the effects of legumes as a protein source on bone quality in broiler are available. In our previous studies, we have demonstrated that partial replacement of SBM with high- and low-tannin raw faba bean seeds in diets up to 20%/30% in starter/grower shows adverse effects on bone development or mechanical strength in broiler chickens [14, 15]. An unambiguously negative effect on musculoskeletal system was observed when lupine seeds were used as a replacement for SBM in broilers diet [39]. On the other hand, chicks on the canola meal diet had longer tibiotarsus and higher bone breaking strength than those on SBM, while the width of bone and general mineral content were similar in both groups [40]. In another study, Wang et al. [4] compared dried distillers grains with solubles diet with meat and bone meal diet and observed no differences in tibiotarsus length, weight, breaking strength, or minerals concentrations [4].

To the best of our knowledge, there is no research on the effects of CPS on the structure of bones in poultry. In this study, the inclusion of CPS as a protein source did not have a significant effect on final body weight of chickens, thus the bones were taken from birds witch matching body weights. It is important, as bone strength, mineralization and histomorphometry are highly correlated with changes in body weight [41, 42]. However, bone weight, length, and Seedor index were significantly increased in the CPS group. Moreover, chickens fed CPS had altered spatial distribution of bone tissue, as indicated both by bone mid-diaphysis cross-sectional area and bone volumetric density.

Bending test showed that bones in the CPS group were characterized by higher values of yield and ultimate load and experienced smaller distortions, both in the region of elastic and plastic deformation, as shown by reduced values of yield and ultimate strain. However, when loads were adjusted to bone shape (yield and ultimate stress) both groups did not differ. Yield and ultimate stresses are better traits than raw bone breaking loads in measuring the effect of treatment on bone strength, as they can correct for bone size [23]. Thus, it can be assumed that overall the dietary protein source probably does not alter bone strength. However, Young modulus which is a material index of bone rigidity, was lower in the SBM group. Thus, observed differences in bones predisposition to deformation under the influence of external forces may be associated with other factors, such as the alterations in bone mineral phase or the structure of the organic matrix. This will be analyzed in the subsequent parts of the discussion.

The quantitative indicators of bone mineralization (mineral density, ash content) were the same in both groups. However, bone mineral composition differed (Table 2). CPS are characterized by higher Sr content than soybean seeds or their by-products, and, as shown by ICP-OES analysis, the Sr content was significantly higher in the CPS group. It has been shown that Sr positively affects cortical bone volume and Sr ions can replace some of the Ca ions in bones of chickens [43]. It was confirmed in our study where the increase of bone volume and Sr/Ca ratio in CPS chicken was observed. Nevertheless, despite the minor differences in bone microelements concentration, the concentration of major macro- and microelements closely related with bone growth (P, Zn, Cu) was the same in both groups.

Bone growth depends also on the action of chondrocytes from the growth plate cartilage [44]. Recently, is has been shown that the growth plate cartilage of birds with induced dyschondroplasia is characterized by increased hypertrophic zone, and inhibited bone mass-related and bone structure-related parameters [45]. Chickens form the CPS group had heavier, longer and wider tibiotarsus than those of SBM group, with reduced hypertrophic zone and increased ossification zone and total growth plate thickness.

In articular cartilage, CPS adversely affected the thickness of transitional zone containing proteoglycans, which provide hydration and swelling pressure to the tissue, making it more resistant and elastic. The association of collagen-proteoglycan system helps to maintain the shape and stabilize the cartilage. The area of the highest proteoglycan concentration was wider in the SBM group, indicating that substances present in CPS might affect the synthesis of proteoglycans. However, the thickness of the superficial zone (zone I) and shape of the articular cartilage surface were the same in both groups, which suggests that the hydration of the cartilage surface which plays an important role in lubrication and frictional characteristics of articular cartilage [46], was similar in both groups.

Collagen from articular cartilage was also examined using DSC analysis. We have previously used DSC technique to detect structural differences of collagen fibres caused by dietary treatments [18, 30]. In the present study, the denaturation temperatures were unaffected by diet type, indicating that crosslinked collagen was thermally very stable in both examined cartilage tissues. However, different net enthalpies suggested a change in collagen structure. We suggest that a reduction of net enthalpy in the CPS group is caused by morphologically less compacted collagen bundles of thin (immature) collagen fibres, the number of which has been shown to be significantly higher in the CPS group (Fig 6). Thin, more loosely packed fibres of immature collagen need significantly less energy to disintegrate their structure. Also trabecular and compact bone in CPS chickens were characterized by a higher number of immature collagen fibres (Fig 6), demonstrating intensive process of formation of new structures. Therefore, it can be suggested, that due to the fact that immature fibers are not hardened by hydroxyapatite particles, stiffness and elastic properties of compact bone were greater in the CPS group (Fig 2).

We were unable to find other studies in which effects of diets based on legumes as a protein source on trabecular bone histomorphometry in broiler chickens were analyzed; however, it has been shown in rat model studies that dietary protein source affects microarchitecture of trabecular bone [47, 48]. In our study chickens fed CPS had significantly improved trabecular bone structure, as showed by greater real bone volume and trabecular number.

We also aimed to examine whether the dietary protein source could alter bone hydroxyapatite structure. A number of cations of elements, such as Ba, Cd, Co, Fe, Mn, Pb, and Sr can replace calcium ions in hydroxyapatites [49, 50]. The changes of crystallinity or hydroxyapatite crystal domain size influence bone mechanical properties [35, 51, 52]. There are also some recent studies showing that dietary additives can influence bone crystals structure in pigs [53] or rats [22]. As shown by FT-IR and XRD analyses (Fig 3, Table 3) no differences in the degree of mineralization or hydroxyapatite crystallites structure were observed in our study. Thus, despite the fact that the content of Mn, Sr, and Fe was different (Table 2), the hydroxyapatite crystal domain size was the same in both groups.

Bone development depends additionally on the functionality of gastrointestinal tract, availability of digestive enzymes and the amount of absorbed nutrients [54]. Anti-nutritive factors like protease inhibitors, phytates, and non-starch polysaccharides present in unprocessed raw seeds have adverse effects on nutrient digestibility and can lead to a reduction of absorption of Ca, P, or Fe [3, 9, 55–57]. Comparing to chickpea seeds soybean meal contains also more fiber which improves the retention of soluble ash and increases the production of hydrochloric acid, improving the solubility of mineral salts [58]. On the other hand, Wang et al. [4] suggested, that when dietary mineral levels in the diets are formulated to meet dietary requirements, as it was in our study, all diets, irrespective of the type and amount of dietary protein, provide the same type and amounts of minerals for deposition in broiler bones. In our previous studies we have showed that partial replacement of SBM with raw faba beans leads to an increase in the intestinal absorptive surface, which allows to maintain the protein and energetic metabolism on sufficient level to ensure proper bone homeostasis [14, 15]. It has proved that these changes are mediated by the action of gut-bone axis, where alterations in intestine structure beneficially promote bone development [24, 59–61]. The same mechanism may be responsible for observed effects of dietary CPS inclusion on bone development in this study. However, it can be only speculated and the mechanism of this action should be further investigated.

## Conclusions

This is the first study analyzing the effects of dietary inclusion of CPS on bone structure in broiler chickens to such an extent. The beneficial effects of CPS inclusion on bone development and mechanical strength were greater than it could have been expected. It suggests that CPS can be a promising replacement for SBM in broilers feeding in the aspect of animal welfare related to the development of the skeletal system.
